# Formal airway assessment prior to emergency tracheal intubation: a regional survey of usual practice

**DOI:** 10.1186/cc9571

**Published:** 2011-03-11

**Authors:** A Karmali, S Saha, P Patel

**Affiliations:** 1 Imperial College Healthcare NHS Trust, Hammersmith Hospital, London, UK

## Introduction

Formal airway assessment prior to tracheal intubation is one of the core skills taught to trainees in anaesthesia and forms part of routine perioperative practice. In the United Kingdom, anaesthetists perform the vast majority of emergency intubations of critically ill patients. We conducted a survey of usual practice and opinion regarding airway assessment in the emergency setting by trainees in anaesthesia.

## Methods

An online survey tool was used to create a structured questionnaire pertaining to participants' experience of emergency tracheal intubation of critically ill patients in hospital wards, emergency departments and critical care units. This was distributed to trainees in anaesthesia across London. Participants were asked how often they had performed a formal airway assessment and whether they felt this would have changed patients' clinical outcome.

## Results

We received 178 responses from anaesthetists with recent experience of difficult tracheal intubations in critically ill patients. One hundred and fifty had encountered grade III/IV views at laryngoscopy. Interestingly, the frequency of these encounters had no relationship to anaesthetic experience. The mean anaesthetic experience was 4.8 (SD 2.6) years. Table 1 highlights how often individuals performed an airway assessment and shows that the majority (73.4%) felt that a formal airway assessment beforehand would not have changed eventual patient outcome. Situational urgency and patient factors (for example, level of consciousness) were cited as factors limiting respondents' ability to perform an airway assessment.

**Table 1:** 

	Never	Sometimes	Always
Airway assessment?	8 (4.5%)	121 (68%)	49 (27.5%)
Changed outcome?	124 (73.4%)	42 (24.8%)	3 (1.8%)

## Conclusions

Previous studies have highlighted difficulties in formal airway assessment of critically ill patients in the Emergency Department [1]. These difficulties - for example, lack of patients' ability to cooperate with an assessment - are mirrored in our survey. The majority of anaesthetists surveyed felt that formal airway assessment prior to emergency tracheal intubation of critically ill patients would make no difference to patient outcome. This suggests that most of those surveyed would question the usefulness of formal airway assessment in context of these circumstances.
